# Dietary Glycyl-Glutamine Supplementation Improves Growth, Immunity, Antioxidant Capacity, and Apparent Digestibility of Weaned Piglets

**DOI:** 10.3390/ani15172573

**Published:** 2025-09-02

**Authors:** Xi Jiang, Dong Li, Mengli Chen, Jianzhong Li, Xihong Zhou, Xia Xiong, Yulong Yin

**Affiliations:** 1 School of Materials and Chemical Engineering, Hubei University of Technology, Wuhan 430068, China; jiangxi@jasonbiotech.com (X.J.); dongli@hbut.edu.cn (D.L.); 2 Hunan Provincial Key Laboratory of Animal Nutritional Physiology and Metabolic Process, National Engineering Laboratory for Pollution Control and Waste Utilization in Livestock and Poultry Production, Institute of Subtropical Agriculture, Chinese Academy of Sciences, Changsha 410125, China; 3 Laboratory of Animal Nutrition and Human Health, College of Life Sciences, Hunan Normal University, Changsha 410013, China; 4 College of Advanced Agricultural Sciences, University of Chinese Academy of Sciences, Beijing 100049, China

**Keywords:** glycyl-glutamine, piglet, immunity, antioxidant capacity

## Abstract

Glutamine (Gln) supplementation during the weaning period can alleviate stress in piglets. However, free Gln has poor stability and low absorption in the small intestine. Glycyl-glutamine (Gly-Gln), a stable dipeptide form of Gln, has been evaluated as a potential alternative in pig nutrition. This study investigated the effects of Gly-Gln at 0, 0.125%, 0.25%, 0.375%, and 0.50%, as well as a Gly + Gln positive control, on growth performance, intestinal morphology, immunity, antioxidant status, and nutrient apparent digestibility in weaned piglets. The results showed that dietary supplementation with 0.25%, 0.375%, or 0.50% Gly-Gln significantly increased average daily gain, average daily feed intake, and final weight (*p* < 0.05). Linear and quadratic effects (*p* < 0.05) were observed for growth performance indicators, suggesting that moderate supplementation levels yielded optimal benefits. Dietary Gly-Gln supplementation with 0.25%, 0.375%, or 0.50% Gly-Gln significantly increased serum immunoglobulin (IgG, IgA, and IgM), insulin, insulin growth factor 1, growth hormone, and T4 and T3 contents, and decreased IFN-γ and IL-1β contents (*p* < 0.05). Diets supplemented with 0.25, 0.375, or 0.50% Gly-Gln increased total antioxidant capacity and superoxide dismutase content in serum and liver, and decreased MDA content (*p* < 0.05). Compared with the negative control group, dietary supplementation of 0.25%, 0.375%, or 0.50% Gly-Gln significantly increased the mRNA expression of *ZO-1*, *Occludin,* and *Claudin-1* in the jejunum (*p* < 0.05). Furthermore, crude protein digestibility was significantly improved in piglets receiving 0.375% and 0.5% Gly-Gln (*p* < 0.05), with a significant linear relationship between Gly-Gln level and digestibility. In conclusion, 0.25% is the minimum effective dose of Gly-Gln for improving weaning outcomes. Gly-Gln is more effective than equivalent doses of free glycine and glutamine in enhancing growth performance, gut barrier integrity, and nutrient utilization in weaned piglets.

## Simple Summary

This study investigated the effects of glycyl-glutamine (Gly-Gln) supplementation on weaned piglets. The results showed that 0.25%, 0.375%, or 0.50% Gly-Gln supplementation improved growth performance, enhanced serum immunity and antioxidant capacity, and improved the apparent digestibility of nutrients. Additionally, it upregulated the mRNA expression of jejunal tight junction proteins (*ZO-1*, *Occludin*, and *Claudin-1*). These findings collectively confirm that the minimum effective additive dose of Gly-Gln in weaning piglet feed is 0.25%.

## 1. Introduction

Glutamine (Gln) is the most abundant amino acid in the serum and serves as the primary energy substrate for intestinal epithelial cells [1,2]. Under normal physiological conditions, Gln is synthesized by glutamine synthetase and plays a crucial role in maintaining intestinal barrier function, regulating immune response, and maintaining antioxidant balance [3,4,5]. Under stress conditions such as piglet weaning, endogenous Gln synthesis often cannot meet the needs of rapid growth and immune system development. Therefore, exogenous Gln supplementation becomes essential to maintain intestinal health. Previous studies have shown that Gln can promote the proliferation of intestinal epithelial cells, intestinal stem cells, or crypt cells, thereby supporting the repair of damaged intestinal mucosa [6,7,8,9]. Additionally, Gln can modulate gut microflora composition, activate intestinal innate immunity, and suppress the overproduction of inflammatory cytokines [10,11,12].

Weaning in piglets leads to immune suppression, intestinal injury, reduced growth performance, and even increased mortality. Dietary supplementation with 1% Gln has been shown to improve growth performance, reduce oxidative stress in the gut, and promote intestinal morphological repair in weaned piglets [13]. However, the practical application of free Gln is limited due to its instability in acidic environments and poor absorption efficiency in the small intestine [14]. In contrast, dipeptides of Gln are more storage stable and soluble, and can be efficiently transported across the intestinal epithelial cells via the peptide transporter 1 (PepT1), where they release the active free Gln [15,16]. In addition, Gly itself has a certain antioxidant potential, which can further relieve oxidative stress by participating in glutathione synthesis [17]. In recent years, glycyl-glutamine (Gly-Gln) has been shown to have potential in modulating gut microbiota and alleviating weaning stress. Yan’s study found that 0.25% Gly-Gln supplementation in the diet can regulate the intestinal microbial community of piglets and facilitate weaning transition [18]. Xu et al. [19] showed that dietary exogenous Gly-Gln ameliorated intestinal microflora imbalance induced by LPS challenge and enriched obligate anaerobic bacteria, as well as bacteria producing short-chain fatty acids. Nevertheless, there is currently a lack of systematic research on the dose–effect relationship of Gly-Gln, especially as the minimum effective dose at sub-effective and sub-physiological dose levels is still unclear. Therefore, it is necessary to further evaluate the range of biological effects of Gly-Gln in weaned piglets and its mechanism.

The objective of this study was to investigate the effects of dietary Gly-Gln supplementation on growth performance, immunity, antioxidant capacity, and apparent digestibility in weaned piglets, and to determine the minimum effective dose required to achieve beneficial outcomes.

## 2. Materials and Methods

### 2.1. Materials and Reagents

Glycyl-glutamine (purity: 97.4%) was purchased from Hubei Hong Peptide Biotechnology Co., Ltd. (Wuhan, Hubei, China). Glycine and glutamine (purity ≥ 98.5%) were obtained from Zhangjiagang Sup Chemical Co., Ltd. (Zhangjiagang, Jiangsu, China).

### 2.2. Animals and Experimental Diets

The experiment was conducted at the Yong’an Experimental Base of the Institute of Subtropical Agriculture, Chinese Academy of Sciences (ISA), located in Changsha, Hunan Province, China. All animal procedures were approved by the Animal Care and Use Committee of ISA (No. ISA-2023-00-21). A total of 216 piglets (Duroc × [Landrace × Large White]; average initial body weight = 7.21 ± 0.11 kg), weaned at 21 days of age, were obtained from the Yong’an Experimental Base of the Institute of Subtropical Agriculture, Chinese Academy of Sciences. Pigs were blocked by sex and litter and were randomly assigned into 6 groups (6 pens/group; 6 pigs/pen), supplemented with 0 (negative control), 0.125%, 0.25%, 0.375% and 0.5% glycyl-glutamine or 0.084% glycine and 0.166% glutamine (Gly + Gln (GG) group), for 28 days feeding period. The basal diet was formulated to meet the nutrient requirements for weaned pigs (NRC, 2012; Table 1). All pigs had ad libitum access to feed and water throughout the trial. Fresh feed was provided twice daily at 08:00 and 16:00. Piglets were housed in environmentally controlled pens with slatted plastic flooring, and room temperature was maintained between 26 and 28 °C.

Body weight was recorded individually on day 0 (initial), day 14, and day 28 (end of the experiment), after a 12 h fasting period. Average daily gain (ADG) was calculated for the periods of day 0–14, day 14–28, and day 0–28 by dividing the body weight gain by the number of days. Feed intake was measured daily on a pen basis, and average daily feed intake (ADFI) was calculated by dividing the total feed consumed by the number of pigs and days. Feed conversion ratio (feed to gain) was computed as the ratio of ADFI to ADG. Fecal consistency was observed twice daily (morning and afternoon) by trained personnel using a 0–3 scoring system where 0 = normal, 1 = soft, 2 = mild diarrhea, 3 = severe diarrhea. A score ≥ 2 was considered diarrhea [20]. Observers were blinded to treatment allocation. Diarrhea index (%) was calculated as the number of diarrhea pig-days divided by total pig-days × 100.

### 2.3. Organ and Tissue Collection

On day 28, one pig with a body weight closest to the average of each pen was selected (six pigs per group), fasted for 12 h, and then slaughtered at the Experimental Slaughter Facility of the Yong’an Experimental Base following blood collection.

Liver tissues (approximately 2 g) were rapidly excised, snap-frozen in liquid nitrogen, and stored at −80 °C for later analysis of antioxidant capacity, which was conducted within four weeks after collection (*n* = 6 per group). A segment of approximately 10 to 15 cm from the mid-jejunum was excised, which was anatomically located in the middle third of the small intestine. Similarly, a segment of approximately 10–15 cm from the distal ileum, located within 30 cm proximal to the ileocecal junction, was also collected. Each intestinal segment was thoroughly rinsed with precooled sterile saline to remove luminal contents and then divided into two portions. One portion (approximately 1 to 2 cm) from each segment (jejunum and ileum) was fixed in 4% formaldehyde–phosphate buffer and stored at 4 °C for histological analysis; the other portion was used to scrape mucosal cell layers using a sterile glass slide, which were immediately frozen in liquid nitrogen and stored at −80 °C for subsequent RNA extraction and analysis of tight junction gene expression.

### 2.4. Blood Biochemical Index

Blood samples were collected from a total of six pigs per treatment group (one pig per pen) via anterior vena cava puncture using sterile 10 mL vacuum blood collection tubes without anticoagulant. After 2 h, the blood samples were centrifuged at 3000× *g* for 10 min at 4 °C using a refrigerated centrifuge (Xiangyi Centrifuge Instrument Co., Ltd., Changsha, Hunan, China). The resulting serum was carefully separated and stored at −80 °C. All samples were analyzed within two weeks. Serum biochemical indexes were determined by using TBA-120FR (Toshiba, Tokyo, Japan), including total protein (TP), albumin (ALB), urea nitrogen (BUN), alanine aminotransferase (ALT), aspartate aminotransferase (AST), alkaline phosphatase (ALP), creatinine (GREA), glucose (GLU), triglyceride (TG), total cholesterol (CHOL), low density lipoprotein (LDL), high density lipoprotein (HDL) and total bilirubin (BILT3). Immunoglobulin M (IgM), IgG, IgA, IFN-γ, interleukin (IL)-1β, insulin-like growth factor-1 (IGF-1), Insulin, T4, T3, and growth hormone (GH) in serum were analyzed by an ELISA kit (Cusabio Biotech Co., Ltd., Wuhan, Hubei, China).

### 2.5. Intestinal Morphology

The intestinal segment was sliced into 5 µm thick sections using a rotary microtome (Leica Biosystems, Wetzlar, Germany) and dyed with hematoxylin and eosin solution. The villi height and crypt depth were measured using an optical microscope (Olympus Corporation, Tokyo, Japan) at a combined magnification of 40. For each pig, ten well-oriented and intact villi with their associated crypts were measured. A total of six intestinal samples per group were analyzed.

### 2.6. Antioxidant Capacity

The content of glutathione peroxidase (GSH-Px), MDA, superoxide dismutase (SOD), and total antioxidant capacity (T-AOC) in serum and liver were determined according to the instructions of the kit (Nanjing Boyan Biological Technology Co., Ltd., Nanjing, Jiangsu, China). For liver analysis, tissue samples were homogenized in ice-cold PBS (1:9 *w*/*v*) using a homogenizer (Jingxin Industrial Development Co., Ltd., Shanghai, China) and centrifuged at 10,000× *g* for 15 min at 4 °C to obtain supernatant for assays. Protein concentrations were normalized across samples using the Bicinchoninic Acid Assay method to ensure comparable results. All measurements were performed in triplicate with appropriate blank and standard controls.

### 2.7. Real-Time PCR

Total RNA extraction of jejunal and ileal mucosa was performed using TRIZOL reagent (Invitrogen, Carlsbad, CA, USA), and then the first-strand cDNA was synthesized using the cDNA synthesis kit (TaKaRa, Dalian, China). Real-time PCR was performed on QuantStudio 5 Real-Time PCR System (Applied Biosystems, Foster City, CA, USA) for quantitative analysis of *Claudin-1*, *Occludin*, and *Zonula occludens-1* (*ZO-1*) mRNA with *GAPDH* gene as internal reference. All primers were synthesized by TsingKe Biological Technology (Changsha, China), and their sequences are shown in Supplementary Table S1.

### 2.8. Apparent Total Tract Digestibility

Apparent total tract digestibility (ATTD) of nutrients was determined using titanium dioxide (TiO_2_) as an indigestible marker, included in all diets at 0.30% (*w*/*w*) by replacing an equivalent amount of corn. The marker was thoroughly mixed into the diets during feed preparation to ensure homogeneous distribution. An adaptation period of 5 days to the TiO_2_-containing diets was allowed before fecal collection.

Fecal samples were collected directly from the rectum of selected pigs (*n* = 6 per group) twice daily on days 26, 27, and 28, pooled per pig, and stored at −20 °C. Feed samples (approximately 150 g) were collected by the quartering method and stored at −20 °C. All samples were dried at 65 °C to constant weight, ground through a 1 mm screen, and analyzed for TiO_2_ concentration using UV-spectrophotometry. The contents of dry matter, crude protein, and gross energy were also measured. ATTD (%) was calculated using the following formula:

ATTD (%) = [1 − (Ti_F_/Ti_f_) × (N_f_/N_F_)] × 100

where T_F_ and T_f_ are the concentration of TiO_2_, respectively, in feed and feces (g/kg of DM), and N_F_ and N_f_ are the concentration of nutrients, respectively, in feed and feces (g/kg of DM).

### 2.9. Statistical Analysis

Data are expressed as mean. The pooled SEM (*n* = 6 per group) was derived from the residual error term of the ANOVA and represent overall variability across treatments. Data were statistically analyzed using One-way ANOVA and Tukey multiple comparisons. All statistical analyses were conducted using the Statistical Package for Social Science (SPSS for Windows, v19.0, USA). Differences were considered statistically significant when *p* < 0.05. Linear and quadratic regression analyses were performed to evaluate the dose-dependent effects of Gly-Gln supplementation. The linear and quadratic contrasts were constructed using orthogonal polynomials based on equally spaced treatment levels (0, 0.125, 0.25, 0.375, and 0.5%). Statistical significance was considered at *p* < 0.05, and tendencies were noted at 0.05 ≤ *p* < 0.10.

## 3. Results

### 3.1. Effect of Gly-Gln on Growth Performance and Diarrhea Rate of Weaned Piglets

Compared with the negative control group (0% Gly-Gln), supplementation with increasing levels of Gly-Gln significantly improved final body weight, ADG, and ADFI in weaned piglets, with a clear linear trend (*p* < 0.05). Notably, significant quadratic effects were observed for final body weight, ADG (0–28 d), and ADFI (0–28 d), with *p*-values of 0.017, 0.013, and 0.008, respectively. Compared to the Gly + Gln group, the 0.25%, 0.375%, and 0.5% Gly-Gln groups showed greater improvements in final body weight, ADG, and ADFI, with similar or lower diarrhea rates (Table 2).

### 3.2. Effect of Gly-Gln on Serum Biochemical Parameters of Weaned Piglets

There was no significant difference in serum biochemical indexes among the experimental groups (*p* > 0.05), except for HDL, which showed a significant difference across groups (*p* = 0.037; Table 3).

### 3.3. Effect of Gly-Gln on Serum Antibody, Inflammatory Factor, and Hormone Levels of Weaned Piglets

Dietary Gly-Gln supplementation with 0.25%, 0.375%, and 0.5% significantly increased serum levels of immunoglobulin (IgG, IgA, and IgM), IGF-1, Insulin, T3, T4, and GH, while significantly reducing IFN-γ and IL-1β concentrations compared to the negative control (*p* < 0.05; Table 4). However, no significant linear or quadratic trends were detected among the Gly-Gln dosage levels.

### 3.4. Effects of Gly-Gln on Antioxidant Capacity of Weaned Piglets

Compared with piglets not supplemented with Gly-Gln, dietary supplementation with glycine and glutamine, or with 0.25%, 0.375%, and 0.5% Gly-Gln, significantly increased T-AOC and SOD levels in both serum and liver, while significantly reducing MDA concentrations (*p* < 0.05; Table 5). However, no significant linear or quadratic trends were observed with increasing Gly-Gln supplementation levels (*p* > 0.05; Table 5).

### 3.5. Effects of Gly-Gln on Intestinal Morphology of Weaned Piglets

There were no significant differences in jejunum and ileum morphology among the test groups (*p* > 0.05; Table 6).

### 3.6. Effects of Gly-Gln on Expression of Intestinal Tight Junction Protein in Weaned Piglets

**Figure 1:**
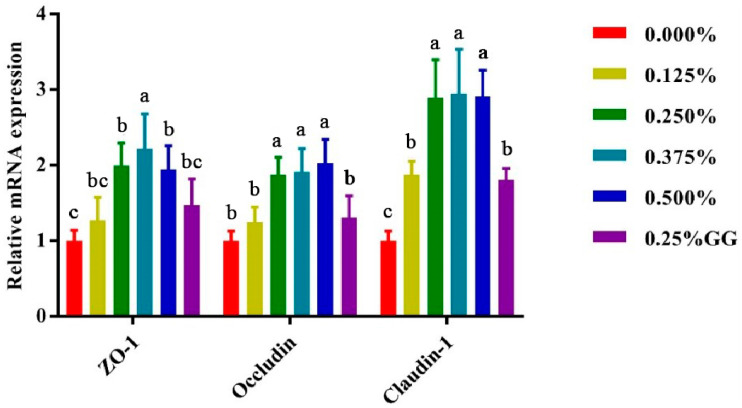
Effect of Gly-Gln on jejunal expression of intestinal tight junction protein of weaned piglets. The expression of *ZO-1*, *Occludin*, and *Claudin-1* was detected by Real-time PCR. Relative expression level was calculated. GAPDH was used as internal control. Data are presented as means ± SEM (n = 6). Different superscript letters (a, b, c) within the same row indicate significant differences between treatment groups (*p* < 0.05). Treatment groups: 0.000% = negative control; 0.125% = 0.125% Gly-Gln; 0.250% = 0.125% Gly-Gln; 0.375% = 0.375% Gly-Gln; 0.500% = 0.500% Gly-Gln; 0.25% GG = 0.25% Gly + Gln; *ZO-1* = zonula occludens-1.

**Figure 2:**
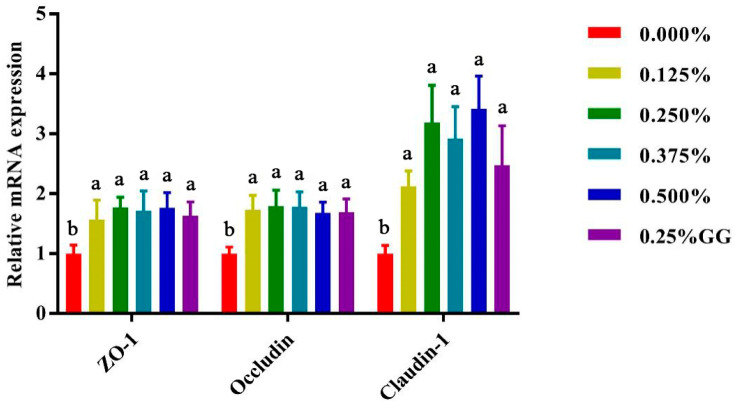
Effect of Gly-Gln on ileal expression of intestinal tight junction protein of weaned piglets. The expression of *ZO-1*, *Occludin*, and *Claudin-1* was detected by Real-time PCR. Relative expression level was calculated. *GAPDH* was used as internal control. Data are presented as means ± SEM (*n* = 6). Different superscript letters (a, b) within the same row indicate significant differences between treatment groups (*p* < 0.05). Treatment groups: 0.000% = negative control; 0.125% = 0.125% Gly-Gln; 0.250% = 0.125% Gly-Gln; 0.375% = 0.375% Gly-Gln; 0.500% = 0.500% Gly-Gln; 0.25% GG = 0.25% Gly + Gln; *ZO-1* = zonula occludens-1.

As shown in Figure 1 and Figure 2, dietary supplementation with 0.25% to 0.5% Gly-Gln significantly upregulated the expression of tight junction genes (*ZO-1*, *Occludin*, and *Claudin-1*) in both the jejunum and ileum of weaned piglets. Among them, *Claudin-1* exhibited the most pronounced increase in both intestinal segments, with expression levels nearly tripled in the 0.25% and 0.5% Gly-Gln groups compared to the control. While *ZO-1* and *Occludin* also showed significant increases, the effect was more notable in the jejunum. The Gly + Gln group showed moderate effects but was generally less effective than higher doses of Gly-Gln (*p* < 0.05).

### 3.7. Effect of Gly-Gln on Apparent Total Tract Digestibility of Weaned Piglets

Compared with the negative control group, supplementation with 0.375% and 0.5% Gly-Gln significantly improved the apparent digestibility of crude protein (*p* < 0.05; Table 7). This improvement exhibited both significant linear (*p* < 0.001) and quadratic (*p* = 0.001) trends (*p* < 0.05; Table 7). No significant trends were observed for dry matter or total energy digestibility.

## 4. Discussion

Weaning is one of the most stressful events in the early life of piglets, often accompanied by a decrease in feed intake, immune function suppression, and damage to the intestinal barrier [21]. Under normal and stable conditions, pigs can synthesize enough endogenous glutamine to meet their own needs [22]. However, during stress states such as weaning or diarrhea, the rate of synthesis is often insufficient, and additional dietary supplementation is required [23]. The results of this study show that the addition of 0.25% or more Gly-Gln to the diet can significantly improve the growth performance, immune response, and intestinal health of weaned piglets.

The observed linear and quadratic trends in BW, ADG, and ADFI indicate that Gly-Gln exerts dose-dependent effects, with moderate levels (0.25–0.375%) yielding optimal benefits. Moreover, Gly-Gln supplementation showed superior efficacy compared to an equivalent mixture of free glycine and glutamine, suggesting that the dipeptide form may offer improved absorption or metabolic advantages, thereby providing greater support for growth and intestinal health during the weaning transition. Additionally, Gly-Gln supplementation did not significantly affect most serum biochemical parameters, implying no adverse impacts on liver or kidney function, or on glucose and lipid metabolism. These findings collectively support the safety and efficacy of Gly-Gln in weaned piglets at the tested inclusion levels.

Weaning is known to trigger the release of pro-inflammatory cytokines such as IL-1β and IL-2 in piglets, which can redirect nutrients toward immune responses instead of growth, thereby impairing growth performance and feed efficiency [24]. Modulating the release of these cytokines may help mitigate the adverse effects associated with immune stress [25]. Previous research has shown that glycine supplementation can alleviate intestinal inflammation. Ji et al. [26] reported that glycine supplementation reduced jejunal IL-1β, IL-6, and TNF-α levels, while enhancing secretory IgA. In contrast, McConn et al. [27] found that 0.2% glutamine had no significant effect on serum levels of IgG, IgM, IL-1β, IL-6, IL-8, IL-10, or TNF-α in weaned piglets. Consistent with findings from Liu [28], Jiang [29], and Yan [18], the present study demonstrated that dietary Gly-Gln supplementation significantly reduced serum IL-1β and IFN-γ concentrations, whereas equivalent doses of free Gln and Gly had no similar effect—likely due to their lower stability and absorption efficiency. These findings support Gly-Gln as a more effective Gln donor with notable immunomodulatory potential. The observed reductions of pro-inflammatory cytokines suggest a possible inhibition of the NF-κB pathway, which is centrally involved in the inflammatory response and known to be suppressed by both glutamine and glycine under stress conditions [30]. In addition to immune modulation, Gly-Gln also exhibited antioxidant benefits, as evidenced by increased total antioxidant capacity and SOD activity in both serum and liver, along with reduced MDA levels. These improvements may stem from the rapid release of glutamine, serving as a substrate for antioxidant defense mechanisms and glycine’s involvement in glutathione synthesis [17,31], collectively helping to mitigate weaning-induced oxidative stress.

The present results showed that Gly-Gln also significantly increased serum IGF-1, Insulin, T3, T4, and GH hormone levels in piglets. Although free amino acid levels in plasma were not directly measured, considering that Gln and Gly are important substrates for the synthesis of citrulline and arginine [32], it is reasonable to assume that the metabolic process of Gly-Gln may promote arginine production, which indirectly affects the expression of insulin and growth-related hormones. Previous studies have shown that arginine is a potent stimulator of insulin secretion from pancreatic beta cells and growth hormone secretion from the anterior lobe of the pendulous body in mammals, and dietary supplementation with 0.4% l-arginine increases plasma insulin and growth hormone levels in piglets [33].

Glutamine serves as a key substrate for the synthesis of nucleic acids, nucleotides, adenosine triphosphate, and nicotinamide adenine dinucleotide phosphate synthesis2 [34], thereby supporting the proliferation of intestinal epithelial cells, intestinal stem cells, and crypt cells, as well as promoting the repair of damaged intestinal mucosa [6,7,8,9]. However, the reported effects of glutamine on intestinal morphology remain inconsistent. Studies in rats have shown that Gln supplementation can increase villi height and the villi height to crypt depth ratio [35,36]. Similarly, Jiang et al. [28] demonstrated that dietary inclusion of 0.15% Gly-Gln improved villus height and crypt depth ratio in the duodenum and ileum of piglets following LPS challenge at 14 days post-weaning. In contrast, Cabrera and Pluske reported that dietary supplementation of 1% or 2% Gln had no significant effect on intestinal morphology in piglets [37,38]. In the present study, Gly-Gln supplementation did not result in statistically significant changes in villus height or crypt depth in either the jejunum or ileum of weaned piglets. Variation in response may be influenced by factors such as duration of supplementation, health status of the animals, or inherent biological variability.

Intestinal tight junction proteins are essential for maintaining mucosal barrier integrity, and their dysregulation is closely linked to various intestinal disorders. Glutamine, as a primary energy source for intestinal cells, supports cell proliferation and nucleotide biosynthesis, thereby playing a key role in preserving intestinal integrity. However, existing reports on the effects of L-glutamine supplementation on tight junction proteins in animals have yielded inconsistent results, possibly due to differences in experimental conditions or developmental stages. For instance, McConn [27] reported that 0.2% L-glutamine supplementation had no significant effect on the expression of ZO-1 and Claudin-1 in the jejunum of weaned piglets. In contrast, Wang showed that dietary 1% L-glutamine significantly upregulated the expression of Occludin, Claudin-1, ZO-2, and ZO-3 proteins, although ZO-1 was not affected [30]. However, Ewaschuk [39] also observed that glutamine supplementation failed to alter Claudin-1 and Occludin expression in piglets infected with *Escherichia coli*. In the present study, dietary supplementation with 0.25%, 0.375%, and 0.5% Gly-Gln significantly increased the mRNA expression of *ZO-1*, *Occludin*, and *Claudin-1* in the jejunum and ileum of weaned piglets, suggesting a strengthening of the intestinal epithelial barrier. These improvements may be partially mediated via the amino acid-sensitive mammalian target of rapamycin (mTOR) pathway, which plays a central role in sensing cellular nutrient status and regulating various physiological processes. In the gut, activation of the mTOR pathway promotes epithelial renewal, enhances tight junction protein expression, and supports immunoglobulin production, thereby contributing to improved intestinal barrier function and host defense mechanisms in weaned piglets [40].

Dietary supplementation of 0.375% glycyl-glutamine significantly increased the crude protein digestibility of piglets, and the improvement followed both linear and quadratic trends with increasing doses, indicating a dose-dependent enhancement. This effect may be attributed to Gly-Gln’s role in supporting gastrointestinal integrity, thereby promoting better digestion and nutrient absorption in weaned piglets [41]. Similarly, Almeida [42] found that dietary supplementation of commercial products consisting of 1% glutamine and glutamic acid also improved crude protein digestibility in weaned piglets.

Weaning stress is one of the most stressful phases of a pig’s life, and since the use of antibiotics in pig production is limited, the assessment and development of effective alternatives is critical to the health of piglets at this stage. Previous studies have established that supplemental l-glutamine may be effective in improving growth performance and gut health in weaned piglets. However, the effects of the combination of amide and glycine on the health of weaned piglets and its effective additive dose are still unknown.

## 5. Conclusions

The results of this study showed that the minimum effective additive dose of glycyl-glutamine in weaning piglet feed was 2.5 g/kg. Glycyl-glutamine could reduce piglet diarrhea, improve piglet digestibility of nutrients and body immunity, and promote piglet growth. This study provides favorable support for the application of glycyl-glutamine in addressing weaning stress in piglets.

## Figures and Tables

**Table 1:** Ingredients and nutrient composition of experimental diets.

Items	Ingredient, %	Nutrient	Calculated Composition
Corn	38.00	Metabolizable energy, MJ/kg	3.24
Puffed corn	15.00	Crude protein, %	18.52
Soybean	3.00	Crude fat, %	6.21
Expanded soybean	6.00	Total phosphorus, %	0.63
Fermented soybean	13.00	Non phytate phosphorus, %	0.42
Whey powder	12.00	SID Thr, %	0.76
Soybean oil	2.00	SID Trp, %	0.16
Fat powder	1.00	SID Lys, %	1.25
Fish meal	4.00	SID Met, %	0.40
Calcium hydrogen phosphate	1.00		
Mineral feed	0.55		
Salt	0.25		
Carrier	2.82	nutrient	Analyzed composition
Titanium dioxide	0.30	Crude protein, %	19.01
Multiple vitamins ^1^	0.03	Crude fat, %	6.32
Multiple minerals ^2^	0.10	Dry matter, %	89.6
Butylated hydroxyanisole	0.02	Metabolic energy, MJ/kg	3.21
Calcium propionate	0.10	Phosphorus, %	0.59
L-Lysine	0.50		
L-Methionine	0.11		
L-Threonine	0.20		
L-Tryptophan	0.02		

^1^ Provided per kilogram of diet: vitamin A, 10,000 IU; vitamin D_3_, 1000 IU; vitamin E, 80 IU; vitamin K_3_, 2.0 mg; vitamin B_1_, 3 mg; vitamin B_2_, 12 mg; vitamin B_7_, 2.25 mg; vitamin B_12_, 12 mg; niacin, 40 mg; biotin, 0.25 mg; folic acid, 1.6 mg; D-pantothenic acid, 25 mg; choline chloride, 300 mg. ^2^ Provided per kilogram of diet: 150 mg of Fe (FeSO_4_); 100 mg of Zn (ZnO); 30 mg of Mn (MnSO_4_); 25 mg of Cu (CuSO_4_); 0.5 mg of I (KIO_3_); 0.3 mg of Co (CoSO_4_); 0.3 mg of Se (Na_2_SeO_3_); and 4.0 mg of ethoxyquin.

**Table 2:** Effect of Gly-Gln on the growth performance and diarrhea rate of weaned piglets.

Items	Gly + Gln	Gly-Gln								
	0.25%	0	0.125%	0.25%	0.375%	0.5%	SEM	*p*-Value	Linear	Quadratic
Body weight, kg										
0 d	7.23	7.20	7.23	7.18	7.22	7.21	0.04	0.976	0.555	0.730
14 d	10.95 ^bc^	10.84 ^c^	10.90 ^bc^	11.34 ^ab^	11.41 ^a^	11.46 ^a^	0.16	0.015	0.017	0.081
28 d	17.21 ^bc^	16.80 ^c^	17.44 ^bc^	18.22 ^ab^	18.67 ^a^	18.91 ^a^	0.29	<0.001	0.006	0.032
Average daily weight gain, g/d										
0–14 d	265.68 ^bc^	259.72 ^c^	262.10 ^c^	297.22 ^ab^	299.21 ^a^	303.57 ^a^	12.55	0.013	0.019	0.092
14–28 d	447.21 ^cd^	426.01 ^d^	467.51 ^bcd^	491.11 ^abc^	519.22 ^ab^	532.51 ^a^	19.33	0.006	0.005	0.028
0–28 d	356 ^b^	342.91 ^b^	364.85 ^b^	394.16 ^a^	408.91 ^a^	418.17 ^a^	10.28	<0.001	0.005	0.031
Average daily feed intake, g/d										
0–14 d	411.71 ^b^	408.33 ^b^	402.38 ^b^	436.90 ^ab^	455.06 ^a^	457.34 ^a^	15.00	0.016	0.019	0.086
14–28 d	718.65 ^cd^	709.71 ^d^	755.75 ^bcd^	777.77 ^bc^	820.03 ^ab^	850.00 ^a^	22.34	0.001	0.001	0.005
0–28 d	559.03 ^d^	559.03 ^d^	580.95 ^cd^	612.79 ^bc^	635.71 ^ab^	653.67 ^a^	11.27	<0.001	0.002	0.008
Feed to gain, g/g										
0–14 d	1.55	1.57	1.55	1.51	1.51	1.52	0.03	0.488	0.048	0.193
14–28 d	1.62	1.67	1.62	1.59	1.58	1.60	0.04	0.594	0.173	0.456
0–28 d	1.59	1.63	1.60	1.56	1.56	1.57	0.03	0.297	0.131	0.381
Diarrhea rate, %										
0–14 d	20.73 ^ab^	24.60 ^a^	22.52 ^ab^	19.44 ^b^	20.24 ^b^	19.54 ^b^	0.04	0.047	0.221	0.442
0–28 d	16.37	17.56	16.62	13.84	14.29	15.08	0.01	0.180	0.121	0.348

Gly + Gln = the glycine and glutamine mixture group; Gly-Gln = the glycyl-glutamine group. Different superscript letters (a, b, c, d) within the same row indicate significant differences between treatment groups (*p* < 0.05). Except for diarrhea rate (*n* = 36), other parameters were measured from 6 piglets per group.

**Table 3:** Effect of Gly-Gln on serum biochemical parameters of weaned piglets (*n* = 6).

Items	Gly + Gln	Gly-Gln						
	0.25%	0	0.125%	0.25%	0.375%	0.5%	SEM	*p*-Value
TP, g/L	53.01	51.51	53.20	56.11	54.32	51.11	0.85	0.578
ALB, g/L	36.01	34.11	34.94	35.62	34.12	32.93	0.67	0.833
ALT, U/L	84.51	87.03	86.13	87.41	86.54	87.83	3.42	1.00
AST, U/L	162.00	114.33	121.17	122.33	115.67	108.50	7.13	0.304
ALP, U/L	318.72	363.51	404.82	329.01	304.33	266.02	19.17	0.401
BUN, mmol/L	2.26	2.45	1.75	2.77	2.08	1.98	0.13	0.270
CREA, μmol/L	87.02	73.21	76.42	74.04	72.32	75.44	1.99	0.565
GLU, mmol/L	4.65	4.53	5.55	4.60	4.60	3.65	0.19	0.148
TG, mmol/L	0.54	0.52	0.68	0.63	0.55	0.46	0.02	0.051
CHOL, mmol/L	2.48	2.43	2.3	2.60	2.38	2.14	0.05	0.220
LDL, mmol/L	1.52	1.48	1.43	1.56	1.42	1.36	0.04	0.802
HDL, mmol/L	1.05 ^ab^	1.07 ^ab^	1.03 ^ab^	1.13 ^a^	1.06 ^ab^	0.87 ^b^	0.02	0.037
BILT3, μmol/L	1.05	0.88	1.02	1.27	1.22	1.45	0.09	0.555

Gly + Gln = the glycine and glutamine mixture group; Gly-Gln = the glycyl-glutamine group. TP = total protein; ALB = albumin; ALT = alanine aminotransferase; AST = aspartate aminotransferase; ALP = alkaline phosphatase; BUN = blood urea nitrogen; GREA = creatinine; GLU = glucose; TG = triglyceride; CHOL = total cholesterol; LDL = low density lipoprotein; HDL = high density lipoprotein; BILT3 = total bilirubin. Different superscript letters (a, b) within the same row indicate significant differences between treatment groups (*p* < 0.05).

**Table 4:** Effect of Gly-Gln on serum antibody, inflammatory factor, and hormone levels of weaned Piglets (*n* = 6).

Items	Gly + Gln	Gly-Gln								
	0.25%	0	0.125%	0.25%	0.375%	0.5%	SEM	*p*-Value	Linear	Quadratic
IgG, mg/mL	13.24 ^bc^	8.94 ^d^	11.68 ^c^	14.09 ^b^	13.09 ^bc^	13.94 ^a^	0.73	<0.001	0.708	0.574
IgA, μg/mL	512.02 ^b^	368.41 ^c^	410.72 ^c^	582.51 ^a^	574.44 ^a^	536.10 ^a^	20.84	<0.001	0.859	0.895
IgM, mg/mL	10.36 ^bc^	7.09 ^d^	8.37 ^cd^	11.93 ^ab^	11.20 ^ab^	11.65 ^a^	0.74	<0.001	0.886	0.805
IFN-γ, pg/mL	33.82 ^b^	39.12 ^a^	31.03 ^bc^	28.71 ^cd^	26.64 ^de^	24.63 ^e^	1.33	<0.001	0.933	0.961
IL-1β, pg/mL	440.02 ^a^	464.84 ^a^	372.21 ^b^	344.31 ^bc^	286.04 ^cd^	261.43 ^d^	22.52	<0.001	0.859	0.987
IGF-1, ng/mL	336.14 ^cd^	282.12 ^d^	394.42 ^bcd^	424.93 ^ab^	418.22 ^abc^	470.71 ^a^	29.18	0.002	0.945	0.829
Insulin, mIU/L	27.64 ^de^	25.25 ^e^	32.15 ^bc^	33.91 ^b^	29.40 ^cd^	31.32 ^a^	0.93	<0.001	0.501	0.822
T4, pmol/L	19.36 ^bc^	13.23 ^d^	17.40 ^c^	19.91 ^b^	23.24 ^a^	24.09 ^a^	0.87	<0.001	0.964	0.999
T3, pmol/L	8.40 ^b^	5.90 ^d^	7.09 ^c^	8.33 ^b^	10.53 ^a^	10.61 ^a^	0.37	<0.001	0.874	0.987
GH, ng/mL	14.50 ^c^	9.09 ^d^	13.10 ^c^	15.47 ^c^	18.22 ^b^	20.77 ^a^	0.73	<0.001	0.977	0.982

Gly + Gln = the glycine and glutamine mixture group; Gly-Gln = the glycyl-glutamine group. IgG = immunoglobulin G; IgA = immunoglobulin A; IgM = immunoglobulin M; IFN-γ = interferon-γ; IL-1β = interleukin-1β; IGF-1 = insulin-like growth factor-1; T4 = thyroxine; T3 = triiodothyronine; GH = growth hormone. Different superscript letters (a, b, c, d, e) within the same row indicate significant differences between treatment groups (*p* < 0.05).

**Table 5:** Effect of Gly-Gln on antioxidant function of weaned piglets (*n* = 6).

Items	Gly + Gln	Gly-Gln								
	0.25%	0	0.125%	0.25%	0.375%	0.5%	SEM	*p*-Value	Linear	Quadratic
Serum										
GSH-Px, nmol/min/mL	904.76 ^a^	735.89 ^c^	789.91 ^bc^	869.34 ^ab^	915.61 ^a^	952.73 ^a^	36.41	0.002	0.677	0.844
MDA, nmol/mL	21.11 ^bc^	35.11 ^a^	26.14 ^b^	14.83 ^d^	18.11 ^cd^	17.98 ^cd^	1.88	<0.001	0.880	0.883
SOD, U/mL	114.05 ^abc^	64.76 ^d^	73.78 ^cd^	103.87 ^bcd^	120.87 ^ab^	143.78 ^a^	15.49	0.005	0.699	0.924
T-AOC, μmol Trolox/mL	0.21 ^b^	0.20 ^b^	0.24 ^a^	0.25 ^a^	0.25 ^a^	0.23 ^a^	0.01	<0.001	0.542	0.547
Liver										
GSH-Px, nmol/min/g	2105.01 ^ab^	1715.22 ^b^	1876.41 ^bc^	2190.02 ^a^	2264.41 ^a^	2048.34 ^ab^	98.92	0.003	0.627	0.823
MDA, nmol/g	36.41 ^bc^	62.55 ^a^	46.10 ^b^	34.11 ^c^	27.71 ^c^	32.63 ^bc^	4.23	<0.001	0.961	0.713
SOD, U/g	170.00 ^bc^	103.42 ^c^	165.72 ^bc^	187.84 ^b^	182.05 ^b^	293.60 ^a^	21.59	0.001	0.787	0.733
T-AOC, μmol Trolox/g	2.21 ^ab^	1.94 ^c^	2.26 ^a^	2.31 ^a^	2.01 ^bc^	2.24 ^a^	0.07	0.003	0.964	0.823

Gly + Gln = the glycine and glutamine mixture group; Gly-Gln = the glycyl-glutamine group. GSH-Px = glutathione peroxidase; SOD = superoxide dismutase; T-AOC = total antioxidant capacity. Different superscript letters (a, b, c, d) within the same row indicate significant differences between treatment groups (*p* < 0.05).

**Table 6:** Effect of Gly-Gln on intestinal morphology of weaned piglet (*n* = 6).

Items	Gly + Gln	Gly-Gln						
	0.25%	0	0.125%	0.25%	0.375%	0.5%	SEM	*p*-Value
Jejunum								
Villus height	549.15	556.32	583.24	588.24	572.22	555.36	22.76	0.780
Crypt depth	457.37	445.21	482.11	499.22	488.45	477.33	22.67	0.558
VH:CD	1.21	1.28	1.25	1.12	1.21	1.15	0.07	0.557
Ileum								
Villus height	470.21	471.35	500.01	503.44	524.12	510.68	26.62	0.641
Crypt depth	474.33	464.46	506.32	528.23	503.33	508.21	23.29	0.416
VH:CD	1.00	1.08	1.03	0.94	1.09	1.05	0.08	0.804

Gly + Gln = the glycine and glutamine mixture group; Gly-Gln = the glycyl-glutamine group.

**Table 7:** Effect of Gly-Gln on apparent total tract digestibility of weaned piglets (*n* = 6).

Items	Gly + Gln	Gly-Gln								
	0.25%	0	0.125%	0.25%	0.375%	0.5%	SEM	*p*-Value	Linear	Quadratic
Crude protein	83.83 ^ab^	82.94 ^b^	84.51 ^ab^	84.19 ^ab^	85.93 ^a^	86.22 ^a^	0.29	0.002	<0.001	0.001
Dry matter	78.94 ^ab^	80.97 ^a^	79.19 ^ab^	78.41 ^b^	80.78 ^a^	80.16 ^ab^	0.26	0.09	0.269	0.354
Total energy	83.70 ^ab^	85.33 ^a^	84.32 ^ab^	83.10 ^b^	85.18 ^a^	84.68 ^ab^	0.24	0.036	0.344	0.503

Gly + Gln = the glycine and glutamine mixture group; Gly-Gln = the glycyl-glutamine group. Different superscript letters (a, b) within the same row indicate significant differences between treatment groups (*p* < 0.05).

## Data Availability

The data presented in this study are available in the article.

## Supplementary Materials

The following supporting information can be downloaded at: https://www.mdpi.com/article/10.3390/ani15172573/s1, Table S1: Primer sequence.
