# Supplementary material for: Dietary Glycyl-Glutamine Supplementation Improves Growth, Immunity, Antioxidant Capacity, and Apparent Digestibility of Weaned Piglets
Source: Animals (Basel). 2025 Sep 2;15(17):2573. doi: 10.3390/ani15172573 (PMC12427411; doi:10.3390/ani15172573)
Supplement: Supplementary file 1 [file animals-15-02573-s001.zip › animals-3773141-supplementary.pdf]

Supplementary Table S1. Primer sequence.

Gene	Forward primer (5’–3’)	Reverse primer (3’–5’)	Product length, bp
ZO-1	TCAAGGTCTGCCGAGACAAC	ATCACAGTGTGGTAAGCGCA	140
Occludin	CAGGTGCACCCTCCAGATTG	ATGTCGTTGCTGGGTGCATA	167
Claudin-1	AAACCGTGTGGGAACAACCA	CACATGAAAATGGCTTCCCTC	196
GAPDH	GGGCATGAACCATGAGAAGT	AGCACCAGTAGAAGCAGGGA	241

ZO-1 = zonula occludens-1; *GAPDH* = glyceraldehyde-3-phosphate dehydrogenase.
